# Commentary: A method for identifying neoantigens through isolation of circulating tumor cells using apheresis in patients with advanced-stage cancer

**DOI:** 10.3389/fimmu.2025.1713331

**Published:** 2025-11-24

**Authors:** Maxwel A. Abegg

**Affiliations:** Graduate Program in Science, Technology and Health (PPGCTS), Institute of Exact Sciences and Technology (ICET), Federal University of Amazonas (UFAM), Itacoatiara, Brazil

**Keywords:** diagnostic leukapheresis, circulating tumor cells, immunogenic cell death, DAMPs, photodynamic therapy, psoralen/UVA, GM-CSF, CpG

## Introduction and positioning

Kobayashi and colleagues present a careful and timely advance linking diagnostic leukapheresis (DLA) to non-amplified exomes from circulating tumor cells (CTCs), enabling individualized neoantigen inference in advanced cancer (1). Their use of cell-surface vimentin (CSV) to mitigate epithelial cell adhesion molecule (EpCAM) blind spots associated with epithelial–mesenchymal transition (EMT) within standard apheresis workflows is notably pragmatic (2, 3). With reported outputs on the order of 10^4–10^5 CTCs per ~5-L session in some advanced cases, DLA provides a credible substrate for downstream translational hypotheses—while acknowledging substantial inter-patient variability (1, 4, 5). Building on this foundation, we outline a conservative therapeutic hypothesis that prioritizes safety, quality control, and near-term feasibility.

## Novelty and impact

Unlike legacy autologous vaccines derived from surgical tumors (irradiated whole cells or oxidized lysates), this concept leverages freshly captured CTCs to manufacture a patient-specific vaccine under controlled conditions. Two coordinated steps are proposed: (i) a whole-cell CTC product rendered immunogenically dead (immunogenic cell death, ICD) and later reinfused alongside non-intravenous (IV) adjuvants; and (ii) a genomically informed booster—preferably messenger RNA (mRNA)—administered around three to six weeks later (estimated timing), after neoantigen prioritization from the same DLA-purified CTCs. This two-step vaccine concept remains hypothetical and will require staged preclinical work and early clinical feasibility testing. To our knowledge, a CTC-derived ICD vaccine for advanced solid tumors paired with a DLA-enabled neoantigen booster has not been described. The approach may be combined with programmed death-1 (PD-1) blockade, since vaccines alone often underperform in advanced disease; KEYNOTE-942 (mRNA-4157/V940 plus pembrolizumab) supports the principle that vaccine+checkpoint can outperform checkpoint alone for recurrence-related endpoints (6). Clinical experience with extracorporeal photopheresis (ECP) shows that collection–manipulation–reinfusion workflows are well-established (7, 8), and rare-cell microfluidics can help standardize inputs—though access to such high-throughput platforms (e.g., Mishra et al.) is likely more limited than DLA capture as used by Kobayashi et al. (1, 9).

## Whole-cell versus lysate

We favor whole-cell ICD products to preserve intact surface architectures and endogenous damage-associated molecular patterns (DAMPs) [(ecto-calreticulin, high-mobility group box 1 (HMGB1), adenosine triphosphate (ATP)] that together promote dendritic cell (DC) uptake and cross-presentation (10, 11), and to exploit *in situ* DC-programming principles (12). Nevertheless, CTC lysates remain a valid alternative—eliminating any theoretical residual-viability risk—and should be explored in parallel.

## Proposed operational sketch

DLA is performed per institutional standards to obtain a CTC-enriched product (1, 4, 5, 7, 8). Immunomagnetic CD45 depletion plus positive selection using CSV with or without epithelial cell adhesion molecule (EpCAM), and optionally human epidermal growth factor receptor 2 (HER2)/epidermal growth factor receptor (EGFR), can broaden representation across epithelial–mesenchymal states (2, 3). The purified CTC fraction is transferred to a good manufacturing practice (GMP)–appropriate laboratory. First, singulation/anti-aggregation (hydrodynamic or acoustofluidic) disperses clusters and standardizes dose exposure (9). Second, ICD is induced under calibrated conditions—e.g., photodynamic therapy (PDT) or psoralen/ultraviolet A (UVA)—aiming to abolish clonogenicity while preserving/exposing antigenic architecture and releasing DAMPs (7, 10, 11). Third, comprehensive quality control (QC) is performed: sterility/endotoxin; viability <1%; and zero clonogenicity by adequately powered limiting-dilution/soft-agar assays. Only lots meeting all criteria are formulated and cryopreserved, enabling multiple doses and, importantly, an adequate interval (an estimated few weeks) for full QC turnaround prior to first administration. For adjuvants, we recommend non-IV schedules (e.g., short subcutaneous (s.c.) granulocyte–macrophage colony-stimulating factor (GM-CSF) and intradermal (i.d.) CpG oligodeoxynucleotides (CpG) at sentinel sites) to recruit dendritic cells while limiting systemic reactogenicity (12–14).

## Neoantigen booster design

In parallel, the same purified CTC fraction (or its archived DNA/RNA) undergoes whole-exome sequencing and neoantigen prediction; when available, tumor-tissue sequencing can be integrated to mitigate antigenic divergence and broaden coverage. An individualized booster—preferably messenger RNA (mRNA) for breadth/polyclonality (often dozens of epitopes per product)—is administered an estimated three to six weeks after the initial vaccine, with PD-1 blockade per clinical context (1–3, 6).

## Target population and practical yields

This hypothesis is targeted at advanced solid tumors, where DLA yields are more often sufficient and clinical need is high. Feasibility will vary by histology and burden. While 10^4–10^5 CTCs per session are reported in some advanced cases (1, 5), many patients will yield fewer cells; practical contingencies include pooling across sessions, optimizing enrichment/recovery, or pivoting to a lysate rather than a whole-cell product when cell counts are modest. Quantitative dose-finding will be necessary to relate administered cell numbers to immune readouts, noting that historical whole-cell vaccines used per-dose cell counts higher than what many CTC collections may provide.

## Translational gates

Sterility/endotoxin: required per lot; closed, validated workflows.
Non-tumorigenicity: zero clonogenicity is non-negotiable; limiting-dilution/soft-agar assays powered to detect rare survivors.
ICD confirmation: batch-level markers (ecto-calreticulin, high-mobility group box 1 (HMGB1), adenosine triphosphate (ATP)) and optional DC-uptake/cross-presentation assays; preservation of key surface epitopes by flow cytometry (10–12).
Target fidelity: multi-marker capture (CSV ± EpCAM ± HER2/EGFR) to mitigate EMT escape and broaden antigenic representation; integrate tissue data when feasible (2, 3).
Hemocompatibility: device/contacting materials tested per International Organization for Standardization (ISO) 10993–4 panels (15).
Adjuvants: prefer s.c./i.d. regimens; consider biomaterial scaffolds for local recruitment where appropriate (12–14).
Analytics: serial CTC kinetics and immune monitoring (enzyme-linked immunospot (ELISpot)/intracellular cytokine staining (ICS)); optional alignment with CTC-guided paradigms (e.g., Study of Circulating Tumor Cells (STIC-CTC)) (16).

## Feasibility anchors and risk profile

A laboratory-based approach reduces engineering risk versus in-line modules and allows lotting/cryopreservation for repeat dosing. Residual risks include (i) inadvertent reinfusion of viable tumor cells—addressed only by demonstrable zero clonogenicity across phenotypic diversity; (ii) antigenic divergence because CTCs may not capture all intratumoral clones—mitigated by integrating tissue data and using broad mRNA boosters (2, 3, 6); and (iii) variable efficacy in heavily pretreated, immunosuppressed states—mitigated by PD-1 combination and rational patient selection (3, 6). Access to high-throughput microfluidics remains more limited than DLA itself and may constrain early adoption (9). Costs are non-trivial, but reuse of apheresis infrastructure, standardized disposables, and batched QC can render early trials tractable (5, 7, 8, 17, 18).

## Suggested next steps

Preclinical proof-of-concept: syngeneic/xenograft models to test the full flow—CTC surrogates → ICD induction → reinfusion + GM-CSF/CpG → mRNA booster ± PD-1—measuring CD8^+^ breadth, tumor control, and memory (10–14).
Process engineering: SOPs for singulation and illumination dose; validated QC panels (viability, clonogenicity, ICD markers) with clear go/no-go criteria.
Clinical exploration: dose-escalation in high-CTC cohorts (e.g., hormone receptor–positive (HR+)/HER2-negative metastatic breast cancer), PD-1 from day one, and serial CTC/immune readouts aligned to clinical context (6, 16).

## Conclusion

Kobayashi et al. establish a robust route to obtain CTCs suitable for neoantigen discovery at scale in advanced disease (1). Motivated by that advance, we hypothesize a two-step vaccination path: a CTC-derived whole-cell ICD product manufactured under rigorous QC, followed by a personalized neoantigen booster administered after an estimated three to six weeks. Practical limits include substantial inter-patient variability in CTC yield, which may necessitate pooling collections or pivoting to lysate-based formulations in some cases, and the challenge of scaling standardized capture/QC workflows across centers. These constraints argue for initial evaluation in high-yield histologies and the development of shared, multi-center SOPs before broader deployment. If successful, this strategy could provide clinically meaningful benefit in selected advanced-stage settings and may be combined with PD-1 blockade or other systemic therapies. The concept remains a hypothesis and will require disciplined preclinical work and staged early clinical feasibility testing to establish safety, operability, and signals of efficacy.
